# A nanobody-based molecular toolkit for ubiquitin–proteasome system explores the main role of survivin subcellular localization

**DOI:** 10.3389/fbioe.2022.952237

**Published:** 2023-01-20

**Authors:** Hui Miao, Chang Liu, Hao Ouyang, Peiwen Zhang, Yuping Liu, Chen Zhang, Changping Deng, Yunhui Fu, Jinping Niu, Wenyun Zheng, Fang You, Yi Yang, Xingyuan Ma

**Affiliations:** ^1^ State Key Laboratory of Bioreactor Engineering, East China University of Science and Technology, Shanghai, China; ^2^ Department of Hepatology, Yueyang Hospital of Integrated Traditional Chinese and Western Medicine, Shanghai University of Traditional Chinese Medicine, Shanghai, China; ^3^ Shanghai Key Laboratory of New Drug Design, School of Pharmacy, East China University of Science and Technology, Shanghai, China; ^4^ Department of Chemical and Biomolecular Engineering, National University of Singapore, Singapore, Singapore

**Keywords:** survivin subcellular function, nanobody targeted, TRIM21, apoptosis and cell cycle, anti-cancer

## Abstract

Targeted protein degradation is a powerful tool for determining the function of specific proteins nowadays. Survivin is the smallest member of the inhibitor of the apoptosis protein (IAP) family. It exists in the cytoplasm and nucleus of cells, but the exact function of survivin in different subcellular locations retained unclear updates due to the lack of effective and simple technical means. In this study, we created a novel nanoantibody-based molecular toolkit, namely, the ubiquitin–proteasome system (Nb4A-Fc-T2A-TRIM21), that can target to degrade survivin localized in cytoplasmic and cell nuclear by ubiquitinating, and by which to verify the potential roles of survivin subcellular localization. Also, the results showed that the cytoplasmic survivin mainly plays an anti-apoptotic function by directly or indirectly inhibiting the caspase pathway, and the nuclear survivin mainly promotes cell proliferation and participates in the regulation of the cell cycle. In addition, the Nb4A-Fc-T2A-TRIM21 system can degrade the endogenous survivin protein in a large amount by the ubiquitin–proteasome pathway, and the system can provide theoretical support for ubiquitination degradation targeting other endogenous proteins.

## Introduction

Survivin is the smallest member of the inhibitor of the apoptosis protein (IAP) family and has only a single N-terminal (Baculovirus IAP repeat) BIR domain and a C-terminal α-helix. It is a multifunctional protein and plays critical roles in many processes including apoptosis, cell cycle, chromosome movement, angiogenesis, and chemotherapy resistance (Sah et al., 2006; Botto et al., 2011; Fan et al., 2013; Wang et al., 2016). Survivin is found to be abundantly expressed in a variety of human tumors, but almost undetectable in most normal, terminally differentiated tissues (Ambrosini et al., 1997). These features make survivin a promising target for cancer vaccines and therapeutics. In addition to the unique expression pattern, endogenous survivin also has a special localization; it exists in both the cytoplasm and nucleus of cancer cells. Numerous studies have been conducted on the functional and prognostic significance of its dynamic localization (Faccion et al., 2018; Saito et al., 2018). However, the exact function and value of subcellularly located survivin in cell apoptosis and cell division are still unclear.

Our understanding of biomolecular pathways has been rapidly developed through techniques that regulate protein levels. In the past decade, several strategies to investigate the functional mechanism of nuclear and cytoplasmic survivin on a molecular level have been reported, including the inhibition of survivin transcription, inhibition of survivin post-translational modification, survivin-targeted peptide vaccines, and survivin gene therapy (Fenstermaker and Ciesielski, 2014; Aljaberi et al., 2015; Lyu et al., 2018; Pi et al., 2018). All these methods let researchers gain a much deeper understanding of the essential aspects of survivin. However, some serious limitations still exist, such as the poor specificity of small-molecule inhibitors, off-target effects, and cytokine storms. On the other hand, an effective posttranslational protein knockdown approach, Trim-Away, was reported recently (Clift et al., 2017). Instead of the aforementioned protein-substrate bound and DNA (or RNA) targeting, this method is based on antibody–antigen recognition. By combining ubiquitin ligase and the Fc receptor TRIM21, Trim-Away is capable of rapidly depleting any endogenous proteins through the ubiquitin–proteasome system (UPS), with a significantly reduced risk of phenotypic compensation.

**FIGURE 1:**
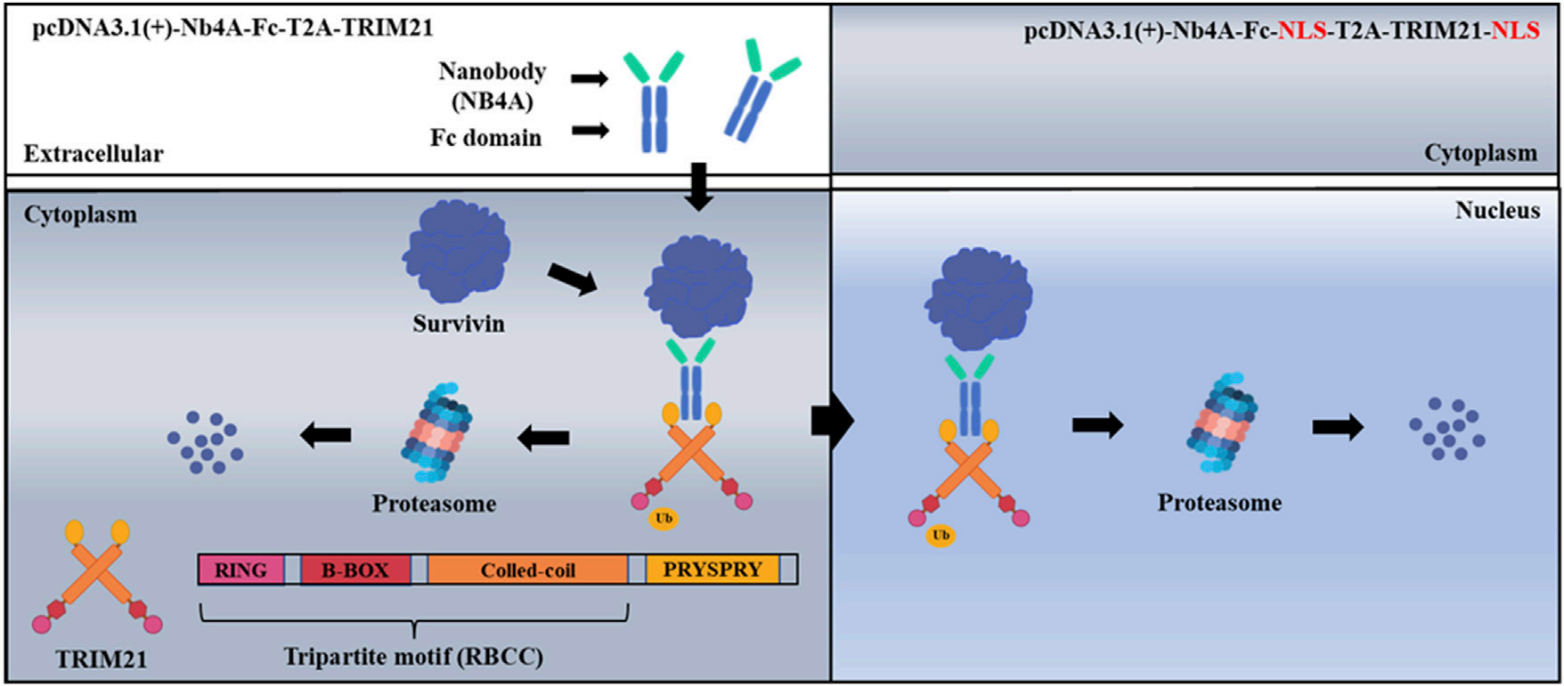
Schematic of ubiquitination targeting survivin in cancer cells. The nanobody Nb4A targets the survivin protein, and the PRYSPRY domain of the TRIM21 carboxyl-terminal binds specifically to the Fc fragment of the fusion protein NB4A-Fc, which leads to a large amount of survivin degradation of cytoplasmic survivin with nuclear survivin *via* the UPS.

We are interested in exploring the molecular mechanism, and prognostic and therapeutic potential of nuclear and cytoplasmic survivin. In the present study, we discovered a nanobody, Nb4A, with high affinity and specificity for survivin, which showed certain anti-tumor activity (Zhang et al., 2016). Nb4A was then used in a Trim-Away-based molecular toolkit to selectively reduce the endogenous survivin level in the cytoplasm or the nucleus of the tumor cells (Figure 1). Our new toolkit provides new mechanistic insight into the multiple roles of survivin in different subcellular localizations and lays a theoretical foundation for survivin as a tumor drug target.

## Materials and methods

### Cell culture

Human breast carcinoma cell line MCF-7 and human normal liver cell line L-02 were purchased from the Type Culture Collection Committee of the Chinese Academy of Sciences (Shanghai, China). MCF-7 and L-02 cells were cultivated in Dulbecco’s modification of Eagle’s medium (DMEM) (Gibco, Germany) with 10% fetal bovine serum (FBS) (Sigma, United States), 100 µ/ml ampicillin, and 100 mg/ml streptomycin. Cells were incubated at 37°C in a humidified atmosphere (5% CO_2_).

### Design and construction of recombinant plasmids

First, Nb4A was fused with the Fc fragment of the antibody, and then the co-expression of Nb4A-Fc and TRIM21 was realized by using the T2A peptide, which constructed the Nb4A-Fc-T2A-TRIM21 toolkit.

pcDNA3.1(+)-Nb4A-Fc-T2A-TRIM21 was constructed by overlapping PCR on the template of the recombinant plasmids pcDNA3.1(+)-Nb4A-Fc and pcDNA3.1(+)-TRIM21 constructed in this experiment. The plasmid was confirmed by HindIII/XhoI digestion and sequenced at Sangon Biotech (Shanghai). Subclone primer pairs: F1: 5’-CCC​AAG​CTT​ATG​CAG​GTG​CAG​CTG​CAG​GAA​AG-3’ (HindIII), R1: 5′-GTGCTGCT GAA​GCC​ATT​GGG​CCA​GGA​TTC​TCC​TCG​AC G​TCA​CCG​CAT​GTT​AGC​AG A​CTT​CCT​CTG​CCC​TCT​CCA​C TG​CCC​TTG​CCG​GGA​GAG​AG-3′, F2: 5′-CTCTCTCCCGGC AA GGG​CAG​TGG​AGA​GGG​CAG​AGG​AAG​TCT​GCT​AAC​AT G​CGG​TGA​CGT​CGA​GGA​GAA​TCC​TGG​CCC​A-3′, and R2: 5′-CCG​CTC​GAG​TCA​ATA​GTC​AGT​GGA​TCC​TTG-3′ (XhoI). pcDNA3.1(+)-Nb4A-Fc-NLS-T2A-TRIM21-NLS was based on pcDNA3.1(+)-Nb4A-NLS-T2A-TRIM21 to fuse the nuclear localization signal NLS (Pro-Lys-Lys-Lys-Arg-Lys-Val) at the carboxyl terminus of the Nb4A-Fc fragment and the TRIM21 fragment. Meanwhile, recombinant plasmids pcDNA3.1(+)-Nb4A, pcDNA3.1(+)-Nb4A-Fc, and pcDNA3.1(+)-TRIM21 were constructed.

### Cell transfection

Lipofectamine^®^ 3000 (cat# L3000015, Thermo Fisher Scientific) was used to transfect different plasmids into the targeted cells. Cells were seeded onto 24- or 6-well plates overnight. When the cells grew to a confluence of 70%–80%, the serum-free and antibiotic-free medium was replaced about 1 h before transfection. Appropriate amounts of the Lipofectamine 3000 reagent and purified DNA were diluted in the Opti-MEM^®^ I medium (cat# 31985, Thermo Fisher Scientific) without serum. Subsequently, an appropriate amount of the P3000 reagent was added to the diluted DNA and incubated for 5 min. The diluted DNA together with the P3000 reagent was then mixed with the diluted Lipofectamine 3000 reagent (1:1 ratio) and incubated for 5 min. The DNA–liposome transfection complex was overlaid onto the cell in DMEM.

### Western blot analysis

To verify the advantage of Nb4A-Fc-T2A-TRIM21 in the targeted degradation of endogenous survivin, the control groups of Nb4A, Nb4A-Fc, and TRIM21 and the negative controls of pcDNA3.1(+) were set up, respectively. The changes in survivin, TRIM21, and FC expression in cells were detected by Western blot, and the expression levels of pro-apoptotic proteins, caspase-3, Bax, and anti-apoptotic proteins Bcl-2 were further detected. Cell cycle proteins play an important role in the process of regulating the cell cycle, and cyclin D1 is one of the types of D cell cycle proteins, which can cycle by activating protein-dependent protein kinases that control the cell cycle process (Kato et al., 1993; Matsushime et al., 1994; Qie and Diehl, 2016). The expression level of cyclin D1 protein after survivin degradation in cancer cells was detected by Western blot to further analyze the regulation of survivin in the cell cycle.

After transfection of different plasmids into MCF-7 cells for 48 h, the cells were collected. Cells were lysed using the Radio-Immunoprecipitation Assay (RIPA) cell lysis reagent (cat# R0010, Solarbio, China) that contained 1 mM phenylmethanesulfonyl fluoride (PMSF). The protein concentration was measured by the method developed by Lowry et al., using bovine serum albumin as standard. Equal amounts of protein were subjected to sodium dodecyl sulfate-polyacrylamide gel electrophoresis (SDS-PAGE) on a 10% polyacrylamide gel. The resolved proteins were transferred onto a polyvinylidene fluoride (PVDF) membrane (cat# BSP0161, PALL, United States), which was then exposed to 5% non-fat-dried milk for 2 h at room temperature before incubation overnight at 4°C with different antibodies [anti-survivin and TRIM21 were purchased from ABclonal Technology (China), and anti-caspase-3, anti-Bax, anti-Bcl-2, and anti-cyclin D1 were all purchased from ProteinTech Group (United States)]. The PVDF membrane was then washed three times with TBST (containing .05% Tween 20) before incubation for 1 h at room temperature with different peroxidase-conjugated secondary antibodies. The immune complexes were finally detected with chemiluminescence reagents by a luminous imaging system (cat# Tanon 5200, Shanghai, China). Protein quantification was performed with ImageJ (1.51 K).

The proteasome inhibitor MG132 was used to verify survivin degradation mediated by the UPS.

Degradation of survivin mediated by Nb4A-Fc-T2A-TRIM21 was verified by the UPS, and the proteasome inhibitor MG132 was used to inhibit the UPS. MG132 can permeate cells and selectively inhibit proteasomes. The different concentrations of MG132 (0, 0.1 μM, 0.2 μM, 0.5 μM, 1 μM, 2 μM, 5 μM, 10 μM, 20 μM, and 50 μM) were determined by the MTT experiment. Then, MG132 concentration determined by MTT was used to intervene in the cells, the changes in survivin expression in the cells were detected by Western blot, and the changes in the apoptosis rate were detected by FC (flow cytometry).

### Cycloheximide (CHX) chase assay

The stability of endogenous survivin was determined by CHX chase assays. Cells were transiently transfected with Nb4A-Fc-TRIM21 for 48 h. Cells were treated with CHX (100 μg/ml) for the indicated times after cells were pre-incubated with MG132 (0.5 μM) or DMSO for 3 h, and then collected and lysed by Western blot.

### Immunoprecipitation assay

Cells were collected and lysed using kits. An equal number of proteins was subjected to immunoprecipitation with an anti-survivin antibody as described in the kits. The immunoprecipitation was separated *via* SDS-PAGE, and the conjugates were detected with anti-ubiquitin and anti-survivin antibodies.

### Analysis of cell apoptosis by FC

The MCF-7 and L-02 cells were treated with recombinant plasmids (pcDNA3.1(+)-Nb4A, pcDNA3.1(+)-Nb4A-Fc, pcDNA3.1(+)-TRIM21, pcDNA3.1(+)-Nb4A-Fc-T2A-TRIM21, and pcDNA3.1(+)-Nb4A-Fc-NLS-T2A-TRIM21-NLS) for 48 h, respectively, and the apoptotic rates were assessed by FC using the Annexin V-fluorescein isothiocyanate (Annexin V-FITC)/propidium iodide (PI) kit (Bio Vision, United States).

### The cell viability assay by MTT

The effects of different plasmids on cell viability were determined by MTT. MCF-7 and L-02 cells were seeded in 96-well plates at 2 × 10^4^ cells/well in 200 μl of DMEM containing 10% FBS. When the cells are at 60% confluence, the cells are treated with different plasmids in a fresh medium for 24 h and 48 h, respectively. Then, 200 μl of 3-(4, 5-dimethylthiazol-2-yl)-2,5-diphenyl-tetrazolium bromide (MTT) (Sigma, United States) (5 mg/ml) was added to each well, and plates were incubated for 4 h at 37°C. The supernatant was aspirated, and 150 μl of DMSO was added to dissolve the formazan crystal formed. The OD (optical density) was recorded at 495 nm with a microplate reader (Biotek, Elx800, United States). The absorbance was used as a measurement of the cell viability, normalized to the cells incubated in the control medium, which were considered 100% viable. The percentage of growth inhibition was equal to [1-(OD of treated/OD of control)] × 100%.

### Analysis of cell cycle by FC

The MCF-7 cells were seeded into a 6-well plate at 5 × 10^5^ cells/ml and cultured until attached. The cells were treated with different plasmids in a fresh medium containing 10% FBS and incubated for 48 h. The cell cycle distribution was evaluated by PI staining of nuclei and FC. The pelleted cells were washed twice with a cold polarization beam splitter (PBS) and then fixed in 70% ice-cold ethanol overnight at −20°C. After washing again, the cells (5 × 10^5^/ml) were re-suspended in 500 μl PBS containing RNase A (50 μg/ml) and PI (50 μg/ml) and incubated at 37°C for 30 min. The fluorescence intensity of PI was analyzed with a FACS (fluorescence-activated cell sorting) flow cytometer and CytExpert software.

### Statistical analysis

Data were expressed as mean ± SD. The significance of differences between groups was evaluated using one-way ANOVA with the LSD (least significant difference) *post hoc* test, and *p* < .05 was considered indicating statistically significant differences.

## Results

### Effects of Nb4A-Fc-T2A-TRIM21 on survivin degradation

**FIGURE 2:**
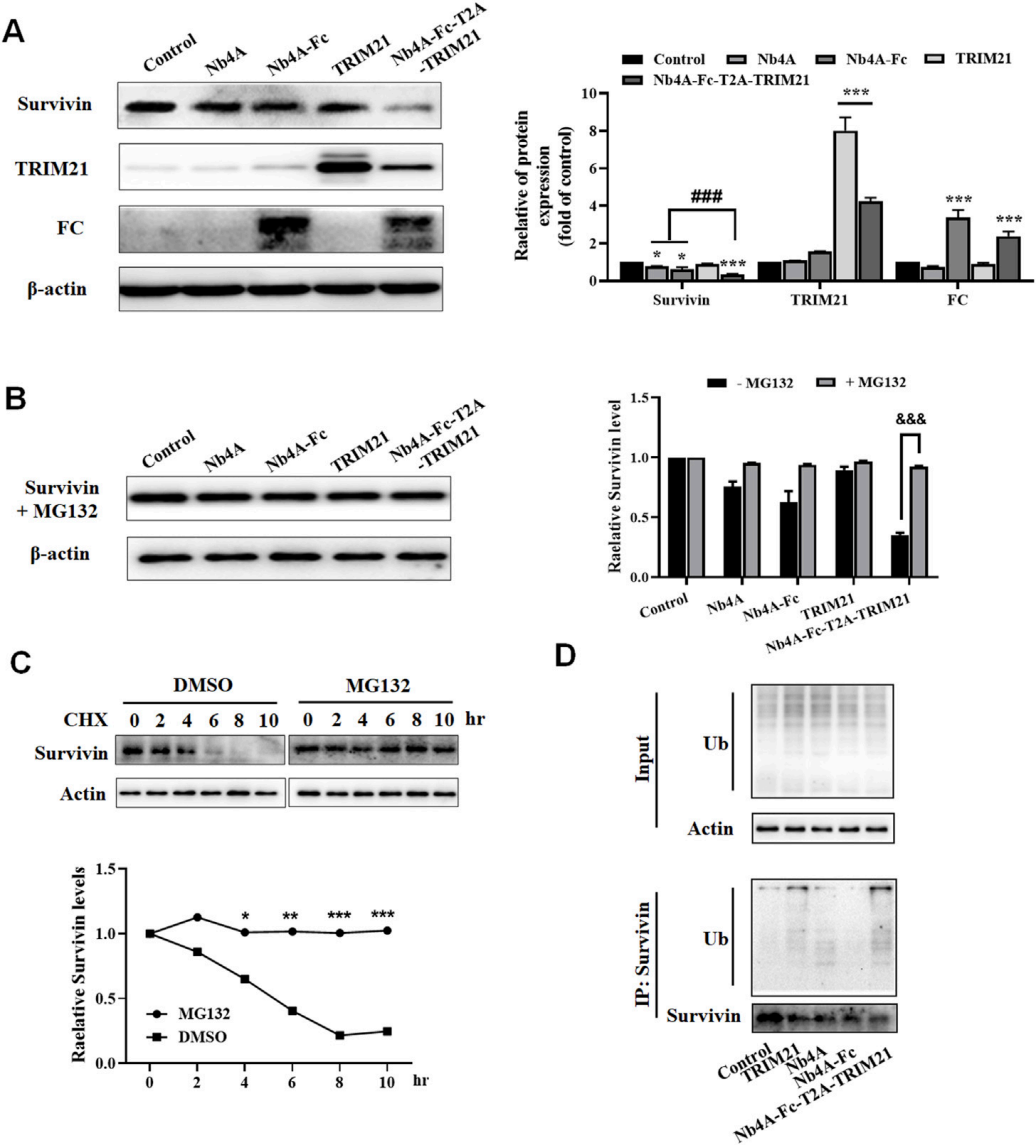
Nb4A-Fc-T2A-TRIM21 promotes the degradation of endogenous survivin. **(A)** MCF-7 cells were transfected with the recombinant plasmids pcDNA3.1(+), pcDNA3.1(+)-Nb4A, pcDNA3.1(+)-Nb4A-Fc, pcDNA3.1(+)-TRIM21, and pcDNA3.1(+)-Nb4A-Fc-T2A-TRIM21 for 48 h. The protein bands of survivin, TRIM21, and FC were normalized to β-actin, and the results are shown right (*n* = 3). **(B)** Protein band of survivin + MG132 was normalized to β-actin, and the results are shown right (*n* = 3). **(C)** MCF-7 cells were treated with CHX (100 μg/ml) for the indicated time, with or without MG132, and then subjected to Western blotting analysis of survivin, and the results are shown below (*n* = 3). **(D)** Ubiquitination assays were performed as described in Materials and Methods. Immunoprecipitation was separated *via* SDS-PAGE, and the conjugates were detected with anti-ubiquitin and anti-survivin antibodies. *p < 0.05, **p < 0.01, and ***p < 0.001 *versus* control, ^###^ *p* < 0.001 *versus* the Nb4A-Fc-T2A-TRIM21 group, and ^&&&^ *p* < 0.001 *versus* the survivin + MG132 group. Data are expressed as mean ± SD.

We designed a scheme for ubiquitin degradation of endogenous survivin at the protein level based on the targeting of nanoantibodies (Figure 1). First, the gene sequences were determined, and the 3D structure of the protein was simulated and predicted (Supplementary Figures S1, S2), which proved the feasibility of this scheme in theory. Then the recombinant plasmids pcDNA3.1(+)-Nb4A, pcDNA3.1(+)-Nb4A-Fc, pcDNA3.1(+)-TRIM21, pcDNA3.1(+)-Nb4A-Fc-T2A-TRIM21, and pcDNA3.1(+)-eGFP-NLS were successfully constructed (Supplementary Figure S3). Among a variety of cells, MCF-7 and L-02, which had strong transfection efficiency and average fluorescence intensity, were selected for later studies (Supplementary Figure S4). After transfection of MCF-7 cells with different plasmids for 48 h, the changes in endogenous survivin, TRIM21, and FC were detected by Western blot. As shown in Figure 2A, compared with the control, the expression of endogenous survivin in the Nb4A group, Nb4A-Fc group, and Nb4A-Fc-T2A-TRIM21 group decreased in varying degrees. The expression level of endogenous survivin in cells of the Nb4A-Fc-T2A-TRIM21 group was the lowest, suggesting that the survivin degradation ability of Nb4A-Fc-T2A-TRIM21 was the strongest. Protein expression of TRIM21 showed TRIM21 expression in the TRIM21 and Nb4A-Fc-T2A-TRIM21 groups, and protein expression of FC showed FC expression in the Nb4A-Fc and Nb4A-Fc-T2A-TRIM21 groups (Figure 2A). The results showed that the exogenous Nb4A-Fc-T2A-TRIM21 could effectively degrade endogenous survivin at the protein level.

### Nb4A-Fc-T2A-TRIM21 mediates survivin degradation through the UPS

After treatment with MG132, the expression of survivin in MCF-7 cells had no significant change compared with the control (Figure 2B). However, there was a significant difference compared with group Nb4A-Fc-T2A-TRIM21 without MG132 (Figure 2B). This shows that proteasome inhibitor MG132 can block the degradation of survivin by Nb4A-Fc-T2A-TRIM21. This is because MG132 can selectively inhibit the proteasome, thus inhibiting the UPS (An and Statsyuk, 2015; Narayanan et al., 2020).

We noticed that the survivin protein level was reduced as early as 6 h. Cycloheximide (CHX) is an inhibitor of eukaryotic protein synthesis. It prevents the degradation of survivin at the protein level, and the WB results show that MG132 together with CHX completely prevented the degradation of survivin (Figure 2C). We next investigated whether survivin protein is degraded *via* ubiquitination in a pathway. In fact, the immunoprecipitation assay results showed that Nb4A-Fc-T2A-TRIM21 degrades survivin protein *via* the ubiquitination pathway (Figure 2D). These data suggest that Nb4A-Fc-T2A-TRIM21 can mediate survivin ubiquitin through the UPS.

### Pro-apoptotic effect of Nb4A-Fc-T2A-TRIM21

**FIGURE 3:**
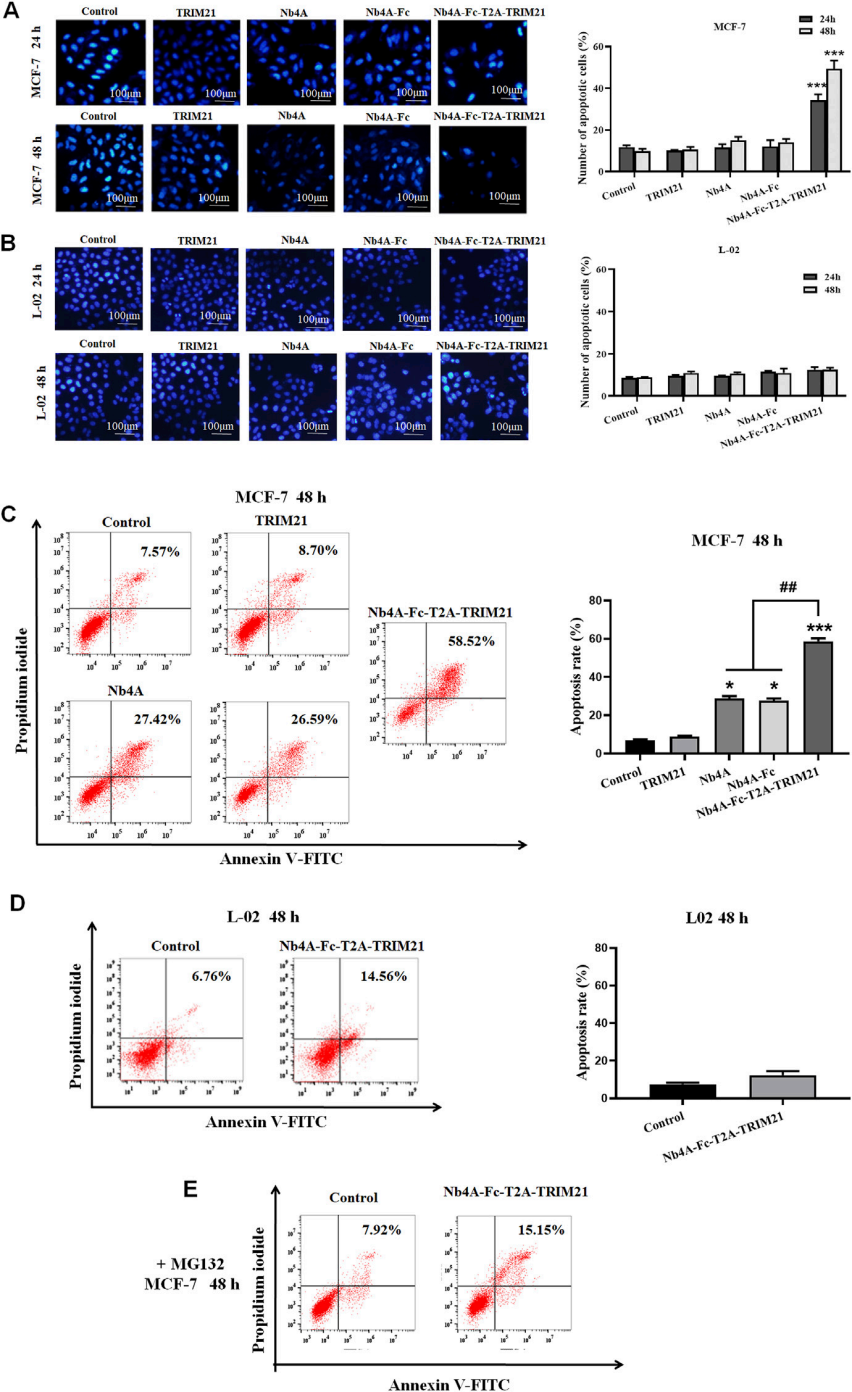
Pro-apoptotic effect of Nb4A-Fc-T2A-TRIM21. **(A)** Using Hoechst 33258 staining, the results of transfection of recombinant plasmids pcDNA3.1(+), pcDNA3.1(+)-Nb4A, pcDNA3.1(+)-Nb4A-Fc, pcDNA3.1(+)-TRIM21, and pcDNA3.1(+)-Nb4A-Fc-T2A-TRIM21 into MCF-7 cells for 24 h and 48 h, and the total percentage of apoptotic cells are shown right (*n* = 3). **(B)** Hoechst 33258 staining was used to transfect L-02 cells with different recombinant plasmids for 24 h and 48 h (bar = 100 μm), and the total percentage of apoptotic cells are shown right (*n* = 3). **(C)** MCF-7 cells were transfected with different recombinant plasmids for 48 h. The apoptosis rate was detected by FC, and the total percentage of apoptotic cells is shown right. **(D)** L-02 cells were transfected with recombinant plasmid pcDNA3.1(+)-Nb4A-Fc-T2A-TRIM21 for 48 h. The apoptosis rate was detected by FC, and the total percentage of apoptotic cells is shown right. **(E)** Apoptosis rate of MCF-7 cells transfected with different plasmids for 48 h was determined by FC with MG132 intervention (*n* = 3). *p < 0.05 and ***p < 0.001 *versus* control and ^##^ *p* < 0.01 *versus* the Nb4A-Fc-T2A-TRIM21 group. Data are expressed as mean ± SD.

After 24 and 48 h of MCF-7 and L-02 transfection, the nuclei of the control group remained normal, and the nuclear edge was intact and smooth. In MCF-7, compared with the control group, the nucleus of the Nb4A-Fc-T2A-TRIM21 group changed slightly 24 h after transfection (Figure 3A). The nucleus became irregular, and there may be a trend toward apoptosis, but there was no significant change in other groups; 48 h after transfection, there were changes in the Nb4A-Fc-T2A-TRIM21 group, and the nucleus was fragmented and irregular. The degree of this change was lower in other groups. In L-02, the nuclear morphology of Nb4A, TRIM21, and Nb4A-Fc-T2A-TRIM21 groups showed no significant change compared with the control (Figure 3B). This suggests that Nb4A-Fc-T2A-TRIM21 can induce different levels of apoptosis in cancer cells compared to normal cells.

FC analysis showed that 48 h after transfection of MCF-7 cells with high expression of survivin, the apoptosis rate of the Nb4A-Fc-T2A-TRIM21 group was the highest, up to 58.52%, which was more than twice that of the Nb4A and TRIM21 groups (Figure 3C). However, 48 h after transfection of L-02 with low expression of survivin, the apoptosis rate of the Nb4A-Fc-T2A-TRIM21 group was only 14.56% (Figure 3D). It is suggested that Nb4A-Fc-T2A-TRIM21 has a strong promoting effect on apoptosis. Under the intervention of MG132, the apoptosis rate of the Nb4A-Fc-T2A-TRIM21 group decreased from 58.52% to 15.15% (Figure 3E). This suggests that Nb4A-Fc-T2A-TRIM21 can mediate survivin ubiquitin through the UPS.

### Targeted degradation of survivin analyzed by immunofluorescence

**FIGURE 4:**
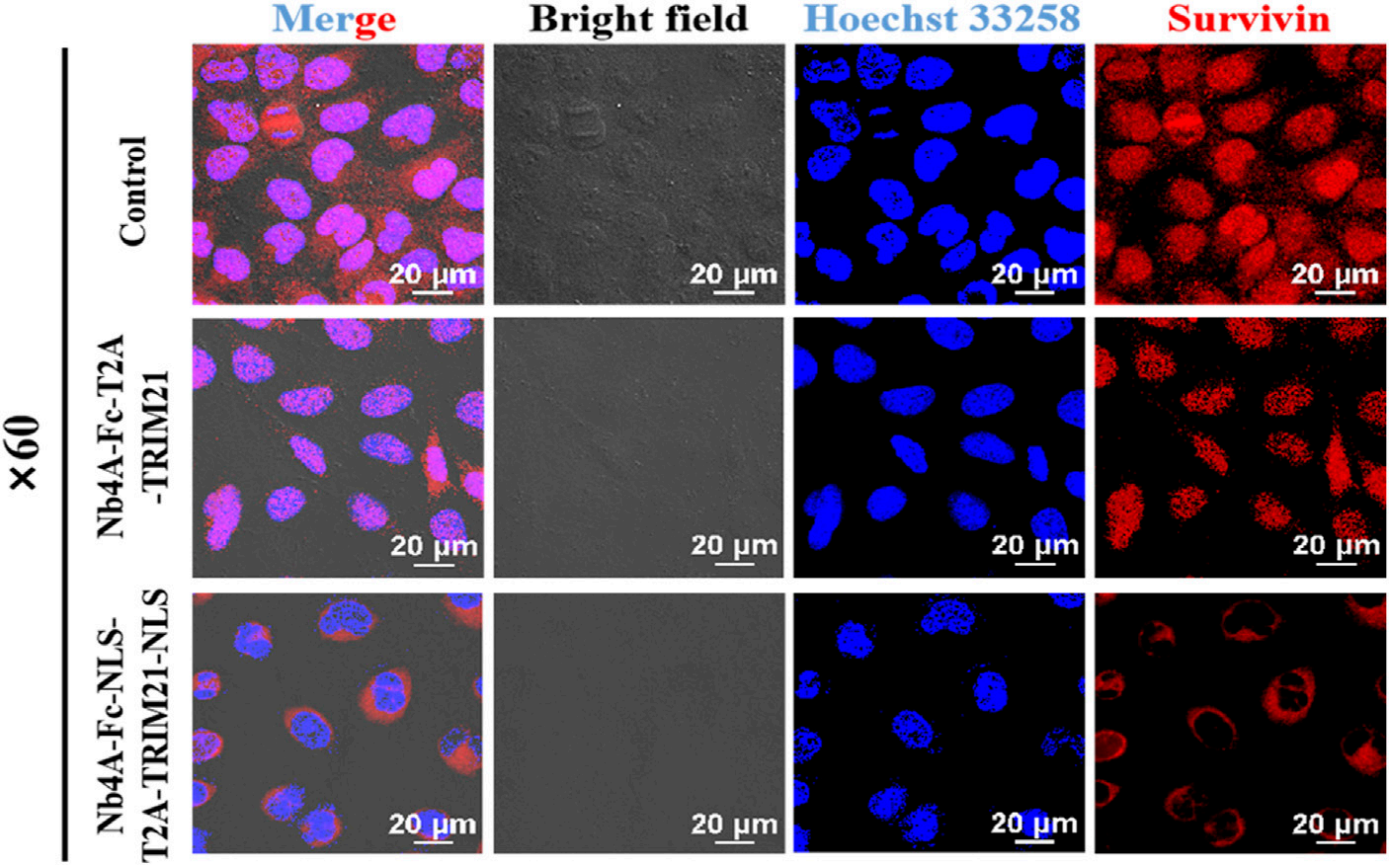
Targeted degradation of survivin analyzed by immunofluorescence. MCF-7 cells were transfected with the recombinant plasmids pcDNA3.1(+)-Nb4A-Fc-T2A-TRIM21 and pcDNA3.1(+)-Nb4A-Fc-NLS-T2A-TRIM21-NLS. After 48 h, targeted survivin degradation was detected by the immunofluorescence assay (bar = 20 μm).

To explore the function of survivin in different subcells (Kachaner et al., 2017), we constructed a plasmid pcDNA3.1(+)-Nb4A-Fc-NLS-T2A-TRIM21-NLS which can realize the nuclear localization of survivin (Supplementary Figure S3, S5). Immunofluorescence results showed that survivin was distributed in both the cytoplasm and nucleus of MCF-7(Figure 4). The cytoplasmic survivin was degraded in the Nb4A-Fc-T2A-TRIM21 group. In the Nb4A-Fc-NLS-T2A-TRIM21-NLS group, nuclear survivin was degraded, but cytoplasmic survivin was not degraded. This suggests that Nb4A-Fc-T2A-TRIM21 can degrade cytoplasmic survivin, while Nb4A-Fc-NLS-T2A-TRIM21 can achieve targeted degradation of nuclear survivin.

### Effects of survivin degradation in different subcells on cell proliferation

**FIGURE 5:**
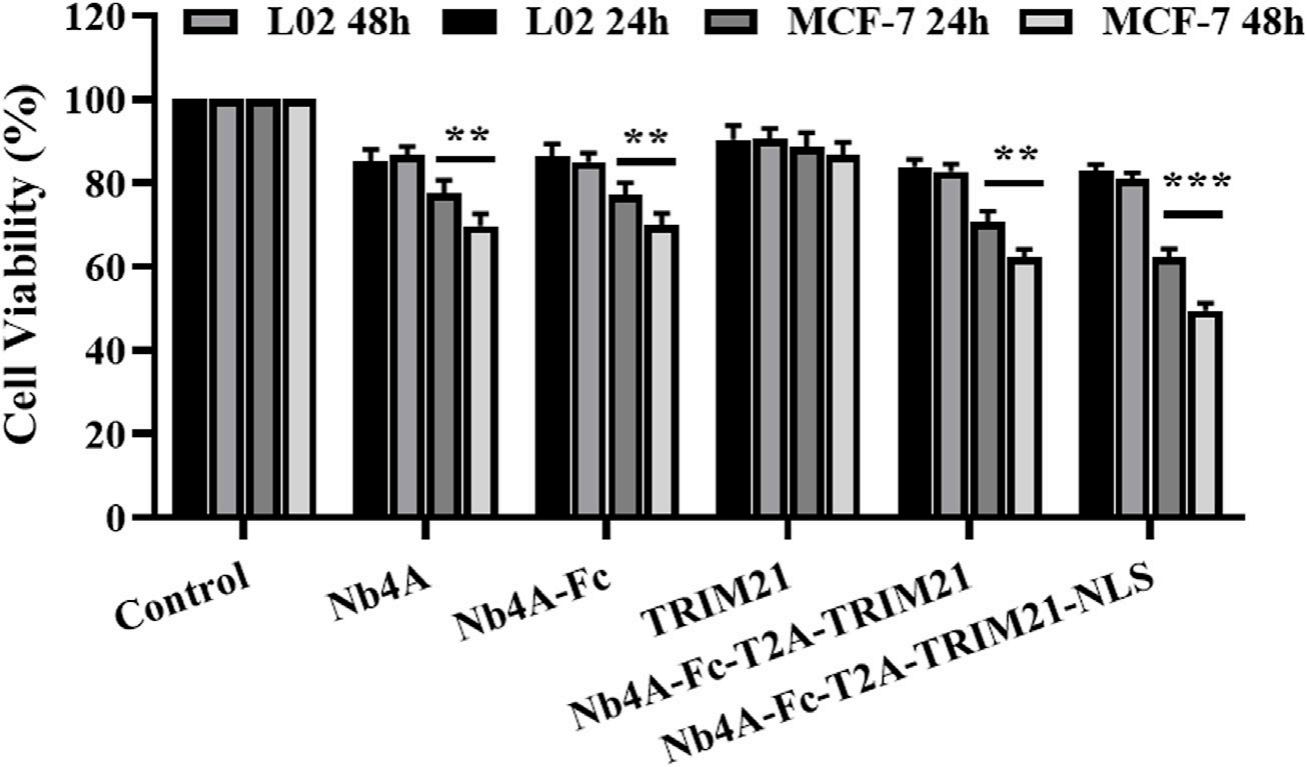
Effects of survivin degradation in different subcells on cell proliferation. The cell viabilities of MCF-7 and L-02 cells were measured by transfecting them with the recombinant plasmids pcDNA3.1(+), pcDNA3.1(+)-Nb4A, pcDNA3.1(+)-Nb4A-Fc, pcDNA3.1(+)-TRIM21, and pcDNA3.1(+)-Nb4A-Fc-T2A-TRIM21 and the plasmid pcDNA3.1(+)-Nb4A-Fc-NLS-T2A-TRIM21-NLS for 24 h and 48 h (*n* = 3). **p < 0.01 and ***p < 0.001 *versus* control. Data are expressed as mean ± SD.

As shown in Figure 5, after 24 h of transfection, Nb4A, Nb4A-Fc, and Nb4A-Fc-T2A-TRIM21 inhibited the MCF-7 cell viability to a certain extent (*p* < 0.01). Nb4A-Fc-NLS-T2A-TRIM21-NLS significantly inhibited the cell viability, among which Nb4A-Fc-NLS-T2A-TRIM21-NLS had the most significant inhibitory effect (*p* < 0.001). Also, the inhibitory effect was time-dependent. After 48 h of transfection, the survival rate of MCF-7 cells was only 49.44% (*p* < 0.001). However, in L-02 cells with low expression of survivin, there was no significant change in cell activity after 24 h and 48 h transfection compared with the control group. The results showed that the degradation of survivin in the nucleus could significantly inhibit the proliferation of cells. Also, this effect is stronger than the inhibition of cytoplasmic survivin degradation.

### Effects of survivin degradation in different subcells on apoptosis

**FIGURE 6:**
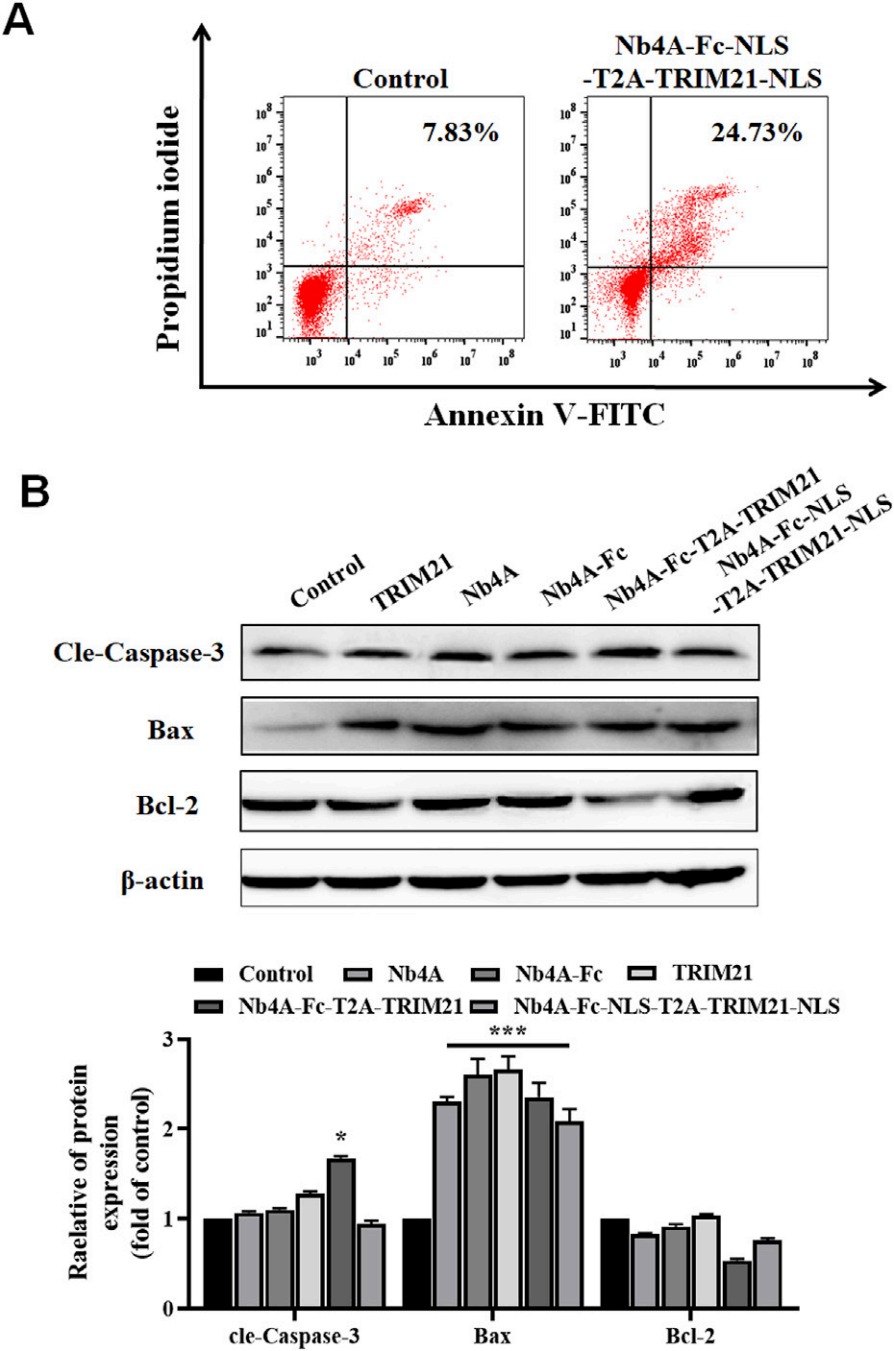
Effects of survivin degradation in different subcells on apoptosis. **(A)** MCF-7 cells were transfected with the recombinant plasmid Nb4A-Fc-NLS-T2A-TRIM21-NLS for 48 h. The apoptosis rate was analyzed by FC. **(B)** Expression levels of apoptosis-related proteins in MCF-7 cells transfected with different recombinant plasmids for 48 h. The expression of cle-caspase-3, Bax, and Bcl-2 was detected in cells. The protein bands of cle-caspase-3, Bax, and Bcl-2 were normalized to β-actin, and the results are shown below (*n* = 3). *p < .05 and ***p < 0.001 *versus* control. Data are expressed as mean ± SD.

FC analysis showed that 48 h after transfection of recombinant plasmid Nb4A-Fc-NLS-T2A-TRIM21-NLS into MCF-7, the apoptosis rate increased significantly to 24.73% (Figure 6A).

As shown in Figure 6B, the expression level of apoptosis-related proteins in MCF-7 cells was transfected with different recombinant plasmids for 48 h. The results of Western blot showed that Nb4A, Nb4A-Fc, TRIM21, Nb4A-Fc-T2A-TRIM21, and Nb4A-Fc-NLS-T2A-TRIM21-NLS could significantly increase the protein expression of Bax, while Nb4A-Fc-T2A-TRIM21 could also significantly increase the protein expression of caspase-3 but had no significant effect on the protein expression of Bcl-2.

### Effects of survivin degradation in different subcells on cell cycle

**FIGURE 7:**
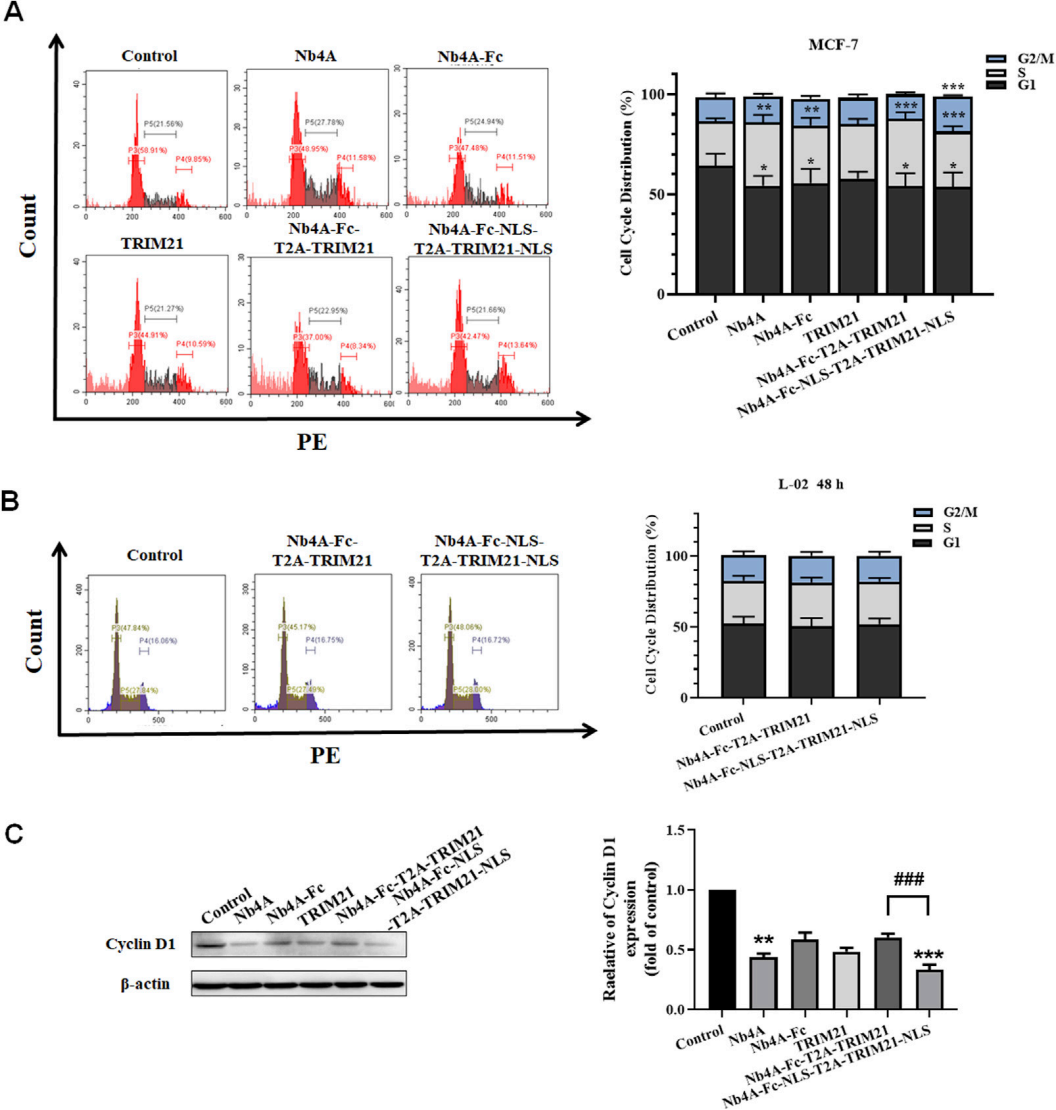
Effects of survivin degradation in different subcells on cell cycle. Cell cycle analysis of MCF-7 cells and L-02 cells. **(A)** Cycle distribution of MCF-7 cells was measured by transfecting the recombinant plasmids pcDNA3.1(+), pcDNA3.1(+)-Nb4A, pcDNA3.1(+)-Nb4A-Fc, pcDNA3.1(+)-TRIM21, and pcDNA3.1(+)-Nb4A-Fc-T2A-TRIM21 and the plasmid pcDNA3.1(+)-Nb4A-Fc-NLS-T2A-TRIM21-NLS for 48 h. The tacked column of cycle distribution is shown right. **(B)** Cycle distribution of L-02 cells was measured by transfecting the recombinant plasmids pcDNA3.1(+)-Nb4A-Fc-T2A-TRIM21 and pcDNA3.1(+)-Nb4A-Fc-NLS-T2A-TRIM21-NLS for 48 h. The tacked column of cycle distribution is shown right. **(C)** Cyclin D1 expression was determined in MCF-7 cells. The protein bands of cyclin D1 were normalized to β-actin, and the results are shown right (*n* = 3). *p < 0.05, **p < 0.01, and ***p < 0.001 *versus* control and ^###^ *p* < 0.01 *versus* the Nb4A-Fc-NLS-T2A-TRIM21-NLS group. Data are expressed as mean ± SD.

The G1 phase proportion of MCF-7 cells transfected with different recombinant plasmids for 48 h decreased to different degrees, and the S phase proportion increased to a different degree. The ratio of the G1 phase in the Nb4A-Fc-NLS-T2A-TRIM21-NLS group decreased from 69.36% to 54.61%, and the S phase increased from 17.05% to 27.85%. The ratio of the G1 phase in the Nb4A-Fc-T2A-TRIM21 group decreased to 54.18%, and the S phase increased to 33.61%. However, the proportion of the Nb4A-Fc-NLS-T2A-TRIM21-NLS group at the G2/M phase increased from 13.59% to 17.54%, while the proportion of Nb4A-Fc-T2A-TRIM21 at the G2/M phase decreased to 12.21% (Figure 7A). These results showed that Nb4A-Fc-NLS-T2A-TRIM21-NLS accelerated the transition from G1 phase to S phase after targeting survivin degradation of the nucleus, and blocked cells in the S and G2 phases to inhibit cell division, while Nb4A-Fc-T2A-TRIM21 did not significantly affect cell cycle progression after targeting survivin degradation of the cytoplasm. Therefore, survivin in the nucleus plays a major role in the survivin regulatory cell cycle.

When L-02 were transfected with pcDNA3.1(+)-Nb4A-Fc-T2A-TRIM21 and pcDNA3.1(+)-Nb4A-Fc-NLS-T2A-TRIM21-NLS for 48 h, the proportion of G1, S, and G2/M phases of L-02 cells did not significantly change (Figure 7B), indicating that the survivin targeted degradation toolkit established in our study did not affect the cell cycle of normal cells L-02 with low expression of survivin.

As shown in Figure 7C, by analyzing the protein expression of cyclin D1, it was found that when the intracellular survivin protein was degraded to different degrees, the expression of cyclin D1 decreased to different degrees. The most significant group was Nb4A-Fc-NLS-T2A-TRIM21-NLS, and cyclin D1 expression in this group was only about half that of the Nb4A-Fc-T2A-TRIM21 group. In addition, the expression of cyclin D1 also decreased significantly in the Nb4A group because Nb4A is a nanobody that can enter the nucleus and bind survivin. Combined with the experiment phenomenon, we can find the survivin downregulation in the cell nucleus decreases cyclin D1 expression, thus inducing cell cycle stagnation and inhibiting cell cycle progression. Also, this result is consistent with the distribution of cell cycle analyzed by FC. Therefore, survivin in the nucleus is mainly playing a role in regulating the cell cycle.

## Conclusion and discussion

Survivin is highly expressed in most cancer cells but hardly in normal cells (Ambrosini et al., 1997; Martínez-García et al., 2019). As an inhibitor of apoptosis, survivin overexpression can inhibit both endogenous and exogenous pathways of apoptosis (Altieri, 2001; Li et al., 2020). At present, it is believed that the mechanism of survivin-inhibiting apoptosis may be related to caspase (Eckelman et al., 2006; Tsang et al., 2017), but its specific mechanism is still not very clear. Apoptosis is generally mediated by activation of the caspase signaling pathway (Ning et al., 2019). Endogenous apoptosis triggers the activation of members of the Bcl-2 family, which controls Bax/Bak channels and increases the permeability of the mitochondrial membrane. This releases cytoc, which binds to Apaf-1 and activates caspase-9, which in turn activates downstream effector caspase-3, 6, and 7 (Riedl and Salvesen, 2007; Youle and Strasser, 2008). Caspase-3 is a major effector caspase, which is a common downstream effector component of multiple apoptotic pathways, and occupies a central position in the apoptotic process, known as the “death-executing protease.” To clarify the biological function of survivin, we need a method to effectively interfere with the protein expression of survivin.

Interfering with protein expression is the most effective way to explore protein function. DNA modification and RNAi are widely used approaches to inhibit protein expression (Doudna and Charpentier, 2014). However, protein depletion in these methods takes a long time (days to months) to effectively remove protein expression (Toyama et al., 2013). Antibodies were previously used to interfere with the function of proteins (Morgan and Roth, 1988). However, antibodies can only competitively bind to protein functional epitopes, thus inhibiting the function of proteins. At the same time, studies have shown that TRIM21 is an E3 ubiquitin ligase, which has a high affinity with the Fc domain of the antibody (James et al., 2007) and can be fused into the Fc segment of the nanobody to play a role in targeted protein degradation (Ibrahim et al., 2020). As an Antibody RING-Mediated Destruction of Endogenous Protein toolkit seems to show minimal off-target destruction (Doudna and Charpentier, 2014), the choice of targets depends on the specificity of the nanobody. This is the main advantage of nanobody-targeted ubiquitin degradation of survivin because this method can selectively degrade proteins and reduce the off-target effect. Here, we have constructed a toolkit that can quickly degrade the endogenous survivin protein. In this toolkit, Nb4A, a nanobody of survivin screened in our phage library, was used to add an Fc region, and exogenous TRIM21 was introduced to make TRIM21 bind to Nb4A to play the function of targeted degradation of survivin protein. The results showed that Nb4A-Fc-T2A-TRIM21 could not only significantly degrade survivin protein but also induce apoptosis in MCF-7 cells while not affecting normal cells. This is because Nb4A-Fc-T2A-TRIM21 mediates the degradation of survivin through the ubiquitin–proteasome pathway.

Studies have shown that proteins with different subcellular localizations play different roles (Engels et al., 2007). As has been established in glioma cells, nuclear survivin inhibits DNA damage repair, while the cytoplasm promotes it as well as the prognosis of different colorectal cancers depending on the localization (Qi et al., 2009; Maksoud, 2022). Survivin exists not only in the cytoplasm but also in the nucleus. Studies have shown that survivin not only inhibits apoptosis and promotes proliferation but also participates in the regulation of the cell cycle. Different localizations of survivin may play different functions (Engels et al., 2007), but the function of different subcellular localizations of survivin in cells is still unclear. Therefore, we have established a toolkit that uses the nuclear localization signal NLS to enable Nb4A-Fc-T2A-TRIM21 to target the degradation of survivin proteins in the nucleus. Results have shown that different subcellular localizations of survivin play different functions. Survivin in the nucleus can promote cell proliferation and participate in the regulation of the cell cycle, while survivin in the cytoplasm mainly plays the function of anti-apoptosis. This study not only provides a toolkit for targeted degradation of corresponding proteins by nanobodies but also provides a rapid and simple method for further study of the function of survivin in cancer.

However, this study has demonstrated the primary function of cytoplasmic survivin and nuclear survivin, but how this function is achieved still needs to be explored. It has been shown that the different spliceosomes of survivin have different expression patterns and cellular localizations, with survivin-ΔEx3 mainly found in the nucleus and survivin-2B in the cytoplasm (Garg et al., 2016). In addition, survivin is also present in mitochondria, where the first 10 amino acids of the N-terminal end of survivin are a specific sequence targeting mitochondria, and Smac can directly bind to mitochondrial survivin to prevent its release into the cytoplasm, thereby reducing the susceptibility of cancer cells to apoptosis (Dunajová et al., 2016). The function of survivin in mitochondria was not explored in this study because ubiquitous degradation of survivin cannot occur in mitochondria.

## Data Availability

The original contributions presented in the study are included in the article/Supplementary Material; further inquiries can be directed to the corresponding authors.

## Abbreviations

IAP, inhibitor of apoptosis protein; UPS, ubiquitin–proteasome system; DMEM, Dulbecco’s modification of Eagle’s medium; FBS, fetal bovine serum; PMSF, phenylmethanesulfonyl fluoride; PVDF, polyvinylidene fluoride; FC, flow cytometry; FITC, fluorescein isothiocyanate; PI, propidium iodide; MTT, 3-(4, 5-dimethylthiazol-2-yl)-2,5-diphenyl-tetrazolium bromide; PBS, polarization beam splitter; CHX, cycloheximide.
