# Supplementary material for: A nanobody-based molecular toolkit for ubiquitin–proteasome system explores the main role of survivin subcellular localization
Source: Front Bioeng Biotechnol. 2023 Jan 20;10:952237. doi: 10.3389/fbioe.2022.952237 (PMC9895104; doi:10.3389/fbioe.2022.952237)
Supplement: Supplementary file 1 [file Table1.DOCX]

~~
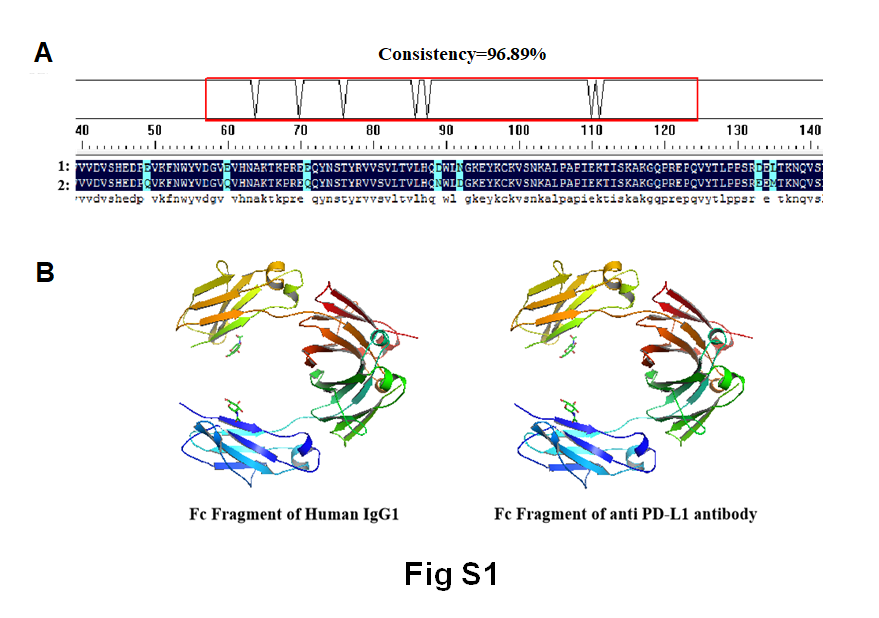
~~**Supplementary materials**

**Figure S1. Consistency analysis of amino acid sequence and three-dimensional structure of the antibody’s Fc fragment. (A)** the consistency analysis of the amino acid sequence of the Fc region of the anti-PD-L1 antibody and the amino acid sequence of the Fc region of human IgG using Muscle software, 1: The amino acid sequence of the Fc region of the human IgG, 2: The amino acid sequence of the Fc region of the anti-PD-L1 antibody. **(B)** Three-dimensional structure simulation.


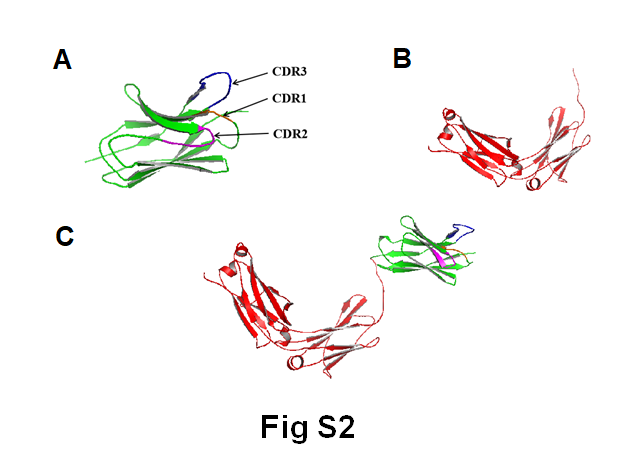


**Figure S2.** **Three-dimensional structure before and after fusion of Nb4A and Fc fragments. (A)** Three-dimensional structure prediction results of Nb4A protein. **(B)** Three-dimensional structure prediction results of Fc fragments. **(C)** 3D structure prediction results of Nb4A-Fc fusion protein.


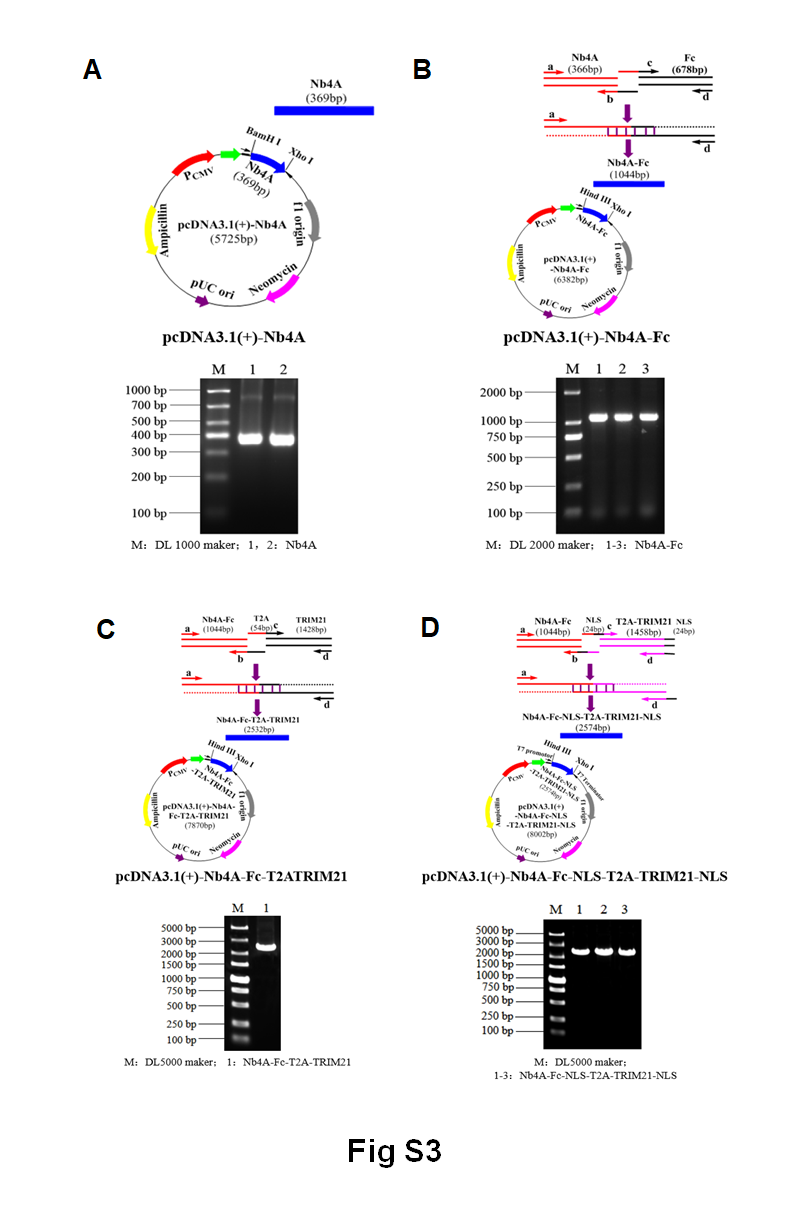


**Figure S3. Schematic diagram of recombinant plasmid construction. (A)** Schematic diagram of constructing the recombinant plasmid pcDNA3.1(+)-Nb4A. Agarose gel electrophoresis of pcDNA3.1(+)-Nb4A via PCR amplification are shown below. **(B)** Schematic diagram of constructing the recombinant plasmid pcDNA3.1(+)-Nb4A-Fc. Agarose gel electrophoresis of pcDNA3.1(+)-Nb4A-Fc via PCR amplification are shown below. **(C)** Schematic diagram of constructing the recombinant plasmid pcDNA3.1(+)-Nb4A-Fc-T2A-TRIM21. Agarose gel electrophoresis of pcDNA3.1(+)-Nb4A-Fc-T2A-TRIM21 via PCR amplification are shown below. **(D)** Schematic diagram of constructing the recombinant plasmid pcDNA3.1(+)-Nb4A-Fc-T2A-TRIM21-NLS. Agarose gel electrophoresis of pcDNA3.1(+)-Nb4A-Fc-T2A-TRIM21-NLS via PCR amplification are shown below.


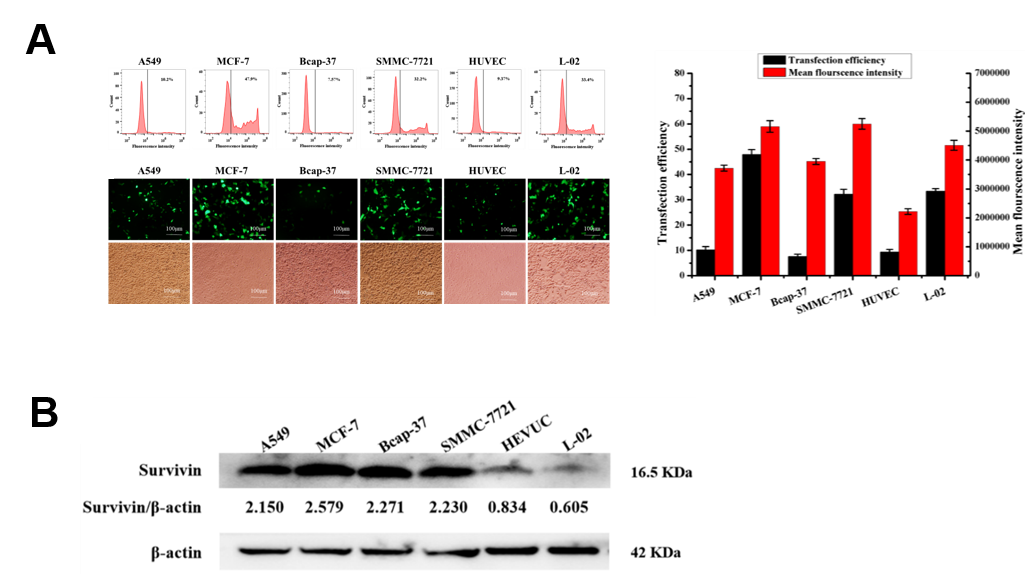


**Figure S4. Cell line optimization. (A)** The eGFP gene was transfected to verify the transfection efficiency and average fluorescence intensity of each cell. Data are expressed as mean ± SD (n=3). **(B)** Expression of endogenous Survivin in cells was determined by western blotting.


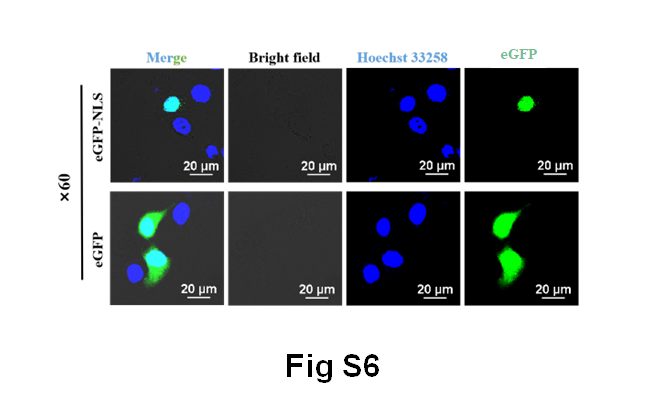


**Figure S5. The localization of eGFP was observed by confocal microscopy.** MCF-7 cells were transfected with the recombinant plasmids pcDNA3.1(+)-eGFP-NLS and pcDNA3.1(+)-eGFP. Localization of eGFP protein was observed by confocal microscope (Bar=20 μm).
